# Deep Learning-Based Detection of Malformed Optic Chiasms From MRI Images

**DOI:** 10.3389/fnins.2021.755785

**Published:** 2021-10-25

**Authors:** Robert J. Puzniak, Gokulraj T. Prabhakaran, Michael B. Hoffmann

**Affiliations:** ^1^Visual Processing Lab, Department of Ophthalmology, Otto-von-Guericke-University, Magdeburg, Germany; ^2^Center for Behavioral Brain Sciences, Otto-von-Guericke-University, Magdeburg, Germany

**Keywords:** chiasmal malformations, albinism, convolutional neural network, CNN, nerve misrouting, misrouting detection, optic chiasm

## Abstract

Convolutional neural network (CNN) models are of great promise to aid the segmentation and analysis of brain structures. Here, we tested whether CNN trained to segment normal optic chiasms from the T1w magnetic resonance imaging (MRI) image can be also applied to abnormal chiasms, specifically with optic nerve misrouting as typical for human albinism. We performed supervised training of the CNN on the T1w images of control participants (*n* = 1049) from the Human Connectome Project (HCP) repository and automatically generated algorithm-based optic chiasm masks. The trained CNN was subsequently tested on data of persons with albinism (PWA; *n* = 9) and controls (*n* = 8) from the CHIASM repository. The quality of outcome segmentation was assessed *via* the comparison to manually defined optic chiasm masks using the Dice similarity coefficient (DSC). The results revealed contrasting quality of masks obtained for control (mean DSC ± SEM = 0.75 ± 0.03) and PWA data (0.43 ± 0.8, few-corrected *p* = 0.04). The fact that the CNN recognition of the optic chiasm fails for chiasm abnormalities in PWA underlines the fundamental differences in their spatial features. This finding provides proof of concept for a novel deep-learning-based diagnostics approach of chiasmal misrouting from T1w images, as well as further analyses on chiasmal misrouting and their impact on the structure and function of the visual system.

## Introduction

The optic chiasm is a key structure in the visual system, where the fate of axons from the retina is decided, such that axons carrying information from the right visual hemifield are guided to the left hemisphere and vice versa. Accordingly, the axons split in the chiasm into two bundles, i.e., axons from the nasal retina that project to the contralateral brain hemisphere (also referred to as “crossing nerves”), and axons from the temporal retina that project to the ipsilateral hemisphere (“non-crossing nerves”). While the normal proportion of axons in the crossing and non-crossing bundle is well established and determined by histological studies to be equal to 53:47, respectively (Kupfer et al., 1967), several congenital disorders are known to affect this arrangement. One example is albinism, where the abnormal development of the visual system (Rebsam et al., 2012) leads to enhanced crossing of the optic nerves at the chiasm resulting in an altered organization of the signal flow in the visual system (Hoffmann et al., 2003). Interestingly, although the altered input to the visual cortex would be expected to fundamentally disrupt signal integration, basic aspects of visual function are preserved, while others (binocular vision, visual acuity, fixation stability) are reduced (Hoffmann and Dumoulin, 2015). This preservation of basic aspects is likely related to processes of cortical plasticity (Hoffmann et al., 2003; Hoffmann and Dumoulin, 2015; Ahmadi et al., 2019) and as such makes human albinism a unique and powerful model of neuroplasticity, granting insights into the structure–function relationship of the visual system. This kind of analysis, however, requires unambiguous and noninvasive mapping of chiasm’s structural features, which is not yet resolved. The first anatomical MRI-based reports (aMRI) of chiasm morphology reported the absence of meaningful anatomical features distinguishing normal and abnormal chiasms (Brodsky et al., 1993). In contrast, two later studies reported differences when comparing chiasm sizes and configurations between controls and people with albinism [PWA; (Schmitz et al., 2003; von dem Hagen et al., 2005)]. Specifically, both studies provided significant evidence of reductions in the width of optic nerves and optic chiasm in PWA, with Schmitz et al. additionally reporting thinner optic nerves and wider angles between optic tracts. Unfortunately, both studies reported group differences but did not explain the aforementioned distinguishing features in the context of diagnostics of chiasmal malformations. Effectively, it is unknown which anatomical features of the chiasm may be employed in an individualized detection of malformations or whether such a detection is possible in the first place. Recently, the application of anatomy-sensitive diffusion MRI (dMRI), capable of estimating the proportion of crossing and non-crossing nerves *via* tractography (Puzniak et al., 2021), has demonstrated chiasmal malformations in albinism at the group level (Ather et al., 2018) with potential for an individualized diagnostic utility (Puzniak et al., 2019). It must be noted, however, that dMRI as compared to aMRI is time consuming at the level of both data acquisition and data analysis. Considering the aforementioned challenges of accurate modeling of chiasmal malformations, it would be of benefit to revisit this issue using models capable of autonomous feature extraction from aMRI data, such as convolutional neural networks [CNNs; (LeCun et al., 1989; Krizhevsky et al., 2012)].

Convolutional neural networks (CNN) are a class of artificial neural networks, i.e., data-driven models inspired by biological systems which are shown to greatly benefit fields relying on computer vision, such as medical imaging (Lundervold and Lundervold, 2019). They are being successfully applied in tasks requiring recognition (segmentation) of brain structures, including the ones involving the optic chiasm (Ibragimov and Xing, 2017; Tong et al., 2018; Chen et al., 2019; Zhu et al., 2019; Duanmu et al., 2020; Mlynarski et al., 2020). This is in particular true for the attempts using MRI data, which have been demonstrated to provide superior contrast and recognition of optic chiasm boundaries compared to other imaging techniques, such as computer tomography (Ibragimov and Xing, 2017; Duanmu et al., 2020). The CNNs, however, are not a universal tool, as their performance is largely dependent on both the quantity and quality of the training data. Consequently, this hinders the development of CNNs in the fields with limited data availability (e.g., due to high data acquisition costs), such as neuroimaging. The above-described limitation is even further augmented in the proposed comparative analysis of normal and abnormal chiasms, where the rarity of albinism [estimated prevalence of albinism equal to 1: 20,000 according to Marçon and Maia (2019)] severely impacts the availability of data from such rare patient groups. These limitations may be counteracted to some degree by known techniques, e.g., transfer learning, allowing to fine-tune existing networks to new data with smaller samples instead of training from scratch. In the present work, we explored the potential of CNNs for the detection of chiasmal abnormalities. For this end, we employed a method that is independent of hardly available, sizable datasets of abnormal chiasms at the expense of interpretability, as discussed in *Limitations*. Specifically, we investigated whether CNNs trained for the purpose of optic chiasm segmentation on control data only, lead to erroneous segmentations for abnormal optic chiasms, e.g., in albinism. Such a differential performance of CNN on normal and abnormal chiasms could be utilized in a quantitative approach for the detection of chiasm abnormalities in albinism and potentially beyond. Currently, albinism diagnosis is based on several morphological and functional features (Hoffmann et al., 2007; Hoffmann and Dumoulin, 2015), with abnormal crossing in the chiasm being one of the major criteria (Kruijt et al., 2018). This is being routinely assessed with functional methods (Hoffmann et al., 2005; von dem Hagen et al., 2008), which are, however, affected by patients’ functional limitations, such as low visual acuity or nystagmus as typical for albinism. Although these limitations would be absent for anatomy-based assessments, the only up-to-date successful reported attempt of an individualized detection of the chiasm abnormalities was achieved with dMRI (Puzniak et al., 2019), which required complex and time-consuming data acquisition and analysis extending beyond the clinical standards. Consequently, the successful CNN-based identification of abnormal chiasms *via* aMRI might provide proof of concept for a novel tool which can be applied to diagnostics, e.g., in albinism.

## Materials and Methods

### Rationale

The objective of this study was to investigate the scope of diagnosing chiasmal malformations using the CNN’s performance as an indicator. For this purpose, we trained a CNN for the segmentation of normal optic chiasms from T1-weighted (T1w) MRI images. This would ideally be achieved by using already developed networks. However, their lacking validation on external datasets, a common issue in the field of DL (Yao et al., 2020), required the development of a custom new network for this purpose and subsequent testing on MRI images of PWA with malformed chiasms. The accuracy of the CNN, determined *via* the comparison of predicted chiasmal masks with previously hand-curated ground-truth masks, is expected to reveal whether representations of malformed chiasms can be learned from control data only. Consequently, the results provide a deeper understanding on whether malformed chiasms are included in the segmentation of representations learned from the control data. This finding is expected to be of value for the clinical diagnostics of malformations, as well as basic research on mechanisms guiding malformation of the chiasms.

### Workflow

**FIGURE 1:**
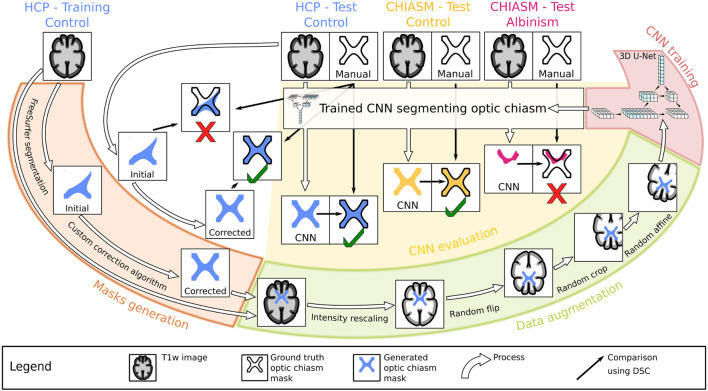
Workflow chart. Graphical illustration of the experiment design. Initially, the training T1w images from the Human Connectome Project (HCP) dataset (top left) were used to generate accurate optic chiasm masks (“Mask generation”; marked by the orange color, left) in a two-step procedure validated on test T1w images. The image–mask pairs were subsequently preprocessed (“Data augmentation”; marked by the green color, bottom right) and used as an input target in the supervised training of the CNN (“CNN training”; marked by the red color, top right). In the final step, the test T1w images from both controls and PWA were used in evaluation of the CNN’s performance (“CNN evaluation”; marked by the yellow color, middle).

This section details the description of the process, the pipeline, and its components, specifically the type of MRI data employed in the training and testing of the CNN, generation of optic chiasm masks by automatic and manual delineation, data augmentation, CNN training, evaluation of CNN on MRI data of controls and PWA, and the metrics used. The graphical overview of the workflow is provided in Figure 1.

### MRI Data

T1w anatomical MRI images of the brain were acquired using 3T MRI. The MRI data came from two separate, publicly available datasets and were used in the (i) training and (ii) evaluation of CNN. Specifically, the CNN was (i) trained on the Human Connectome Project (HCP) dataset (Van Essen et al., 2013), containing nearly 1,200 T1w structural MRIs from control participants, and (ii) tested on the CHIASM dataset (Puzniak et al., in revision)^1^, a repository containing T1w images of patients with rare chiasmal disorders including PWA (*n* = 9) and controls (*n* = 8).

#### HCP Dataset

The CNN was trained on control T1w images (*n* = 1049) from the HCP Dataset—Diffusion MRI 3T 1200 Subjects (S1200) Release (Glasser et al., 2013; Van Essen et al., 2013) downloaded from the brainlife.io platform (Avesani et al., 2019)^2^. As detailed in the PreFreeSurfer pipeline from HCP Minimal Preprocessing Pipelines (Glasser et al., 2013), for each subject, the T1w images acquired with native 0.7-mm isotropic resolution were defaced (Milchenko and Marcus, 2013), aligned to MNI152 template space (rigid-body transformation with 6 degrees of freedom), and corrected for readout distortions (van der Kouwe et al., 2008). The preprocessed images were further resampled to 1.25-mm isotropic resolution to match the resolution of the HCP DWI data. Importantly, the downsampling was also a prerequisite for further segmentation of T1w images with FreeSurfer software.

#### CHIASM Dataset

The performance of the trained CNN was tested on the T1w MRI images of PWA (*n* = 9) and controls (*n* = 8) from the CHIASM dataset (Puzniak et al., in revision, see footnote 1) downloaded from the brainlife.io platform (Avesani et al., 2019)^3^. As preprocessing steps, for each subject, T1w images acquired with native resolution of 0.9 mm were defaced, aligned to Anterior Commissure—Posterior Commissure (ACPC) space, and downsampled to 1-mm isotropic voxel (in order to support FreeSurfer segmentation).

### Optic Chiasm Masks

**FIGURE 2:**
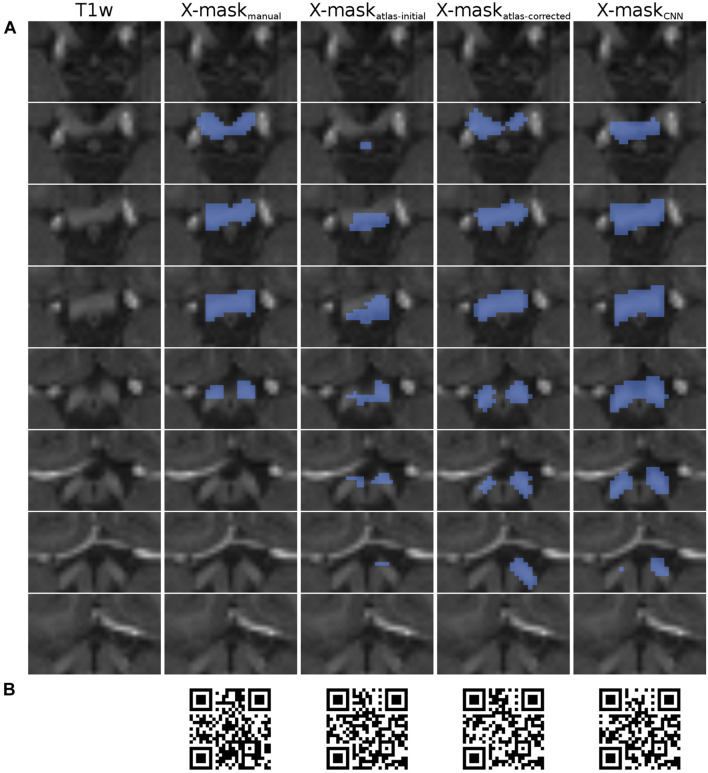
Overview of X-mask from the exemplary HCP dataset (ID 304727). **(A)** Axial slices displaying the optic chiasm region on T1w image (leftmost column). Blue-colored masks superimposed on the T1w slices correspond to, respectively, from left to right: X-mask_manual_, X-mask_atlas–initial_, X-mask_atlas–corrected_, and X-mask_CNN_. The top row displays the most inferior slice, with subsequent rows showing incrementally superior slices. All images are presented in neurological convention. **(B)** QR codes allowing for the inspection of 3D representations of the masks, respectively, from left to right: X-mask_manual_, X-mask_atlas–initial_, X-mask_atlas–corrected_, and X-mask_CNN_.

The T1w MRI images were further used to generate several binary optic chiasm masks through varied approaches. Specifically, this included manually defined ground-truth masks, automatically created masks used for CNN training, and masks of the chiasm computed by the CNN (Figure 2). Although automatically created masks from neuroimaging data are known to be of suboptimal quality (as opposed to ones manually defined by experts), we decided for this approach as it enabled us to analyze a wide range of chiasmal morphologies. This is, in fact, a requisite for the CNNs to robustly identify the well-generalizing features of the chiasm. An overview of the employed masks is provided below, followed by detailed descriptions in the subsequent sections:

• X-mask_manual_—optic chiasm mask defined manually on T1w MRI images.
• X-mask_atlas–initial_—optic chiasm mask created by FreeSurfer’s atlas-based segmentation of HCP training set (*n* = 1049) and CHIASM (*n* = 17) T1w images.
• X-mask_atlas–corrected_—improved optic chiasm masks obtained by correcting X-mask_atlas–initial_ with a custom correction algorithm.
• X-mask_CNN_—optic chiasm mask computed by the CNN from input T1w image. The X-mask_NN_ were generated only for the CHIASM (*n* = 17) dataset and a subset of HCP datasets (*n* = 10; HCP test-controls), which were excluded from CNN’s training and validation procedure (Figure 2A).

Importantly, for the purpose of quality evaluation (see *Computational methods*), the assessed masks were limited only to the axial slices, where the optic chiasm was present, as determined by the X-mask_manual_ (Figure 2A). This step was performed to ensure that the evaluation is focused on the optic chiasm only and is not perturbed by neighboring white matter structures, such as optic nerves and tracts.

#### X-mask_manual_

The X-mask_manual_ were defined in PWA (*n* = 9; CHIASM albinism) and controls (*n* = 8; CHIASM controls) from the CHIASM dataset, and 10 HCP test-controls were excluded from CNN training. Specifically, the delineation was performed by a trained researcher in all the T1w image slices with chiasmal presence, as according to the guidelines detailed in (Puzniak et al., in revision, see footnote 1). The X-mask_manual_ were deemed a ground truth and consequently used as reference for the quality assessment of other masks (Figure 2A).

#### X-mask_atlas–initial_

The X-mask_atlas–initial_ were extracted from the existing atlas-based segmentation of the HCP T1w images (Van Essen et al., 2013) processed according to the HCP FreeSurfer pipeline (Glasser et al., 2013) using FreeSurfer v5.2 (Fischl, 2012). Although such atlas-based masks were successfully used in previous studies aiming to accelerate brain segmentation using CNNs (Fedorov et al., 2017a, b; McClure et al., 2019), our comparison of X-mask_atlas–initial_ with X-mask_manual_ revealed a significantly lower quality of the former (see *Results*), thereby making them (as expected) a suboptimal choice as training data.

#### X-mask_atlas–corrected_

Although X-mask_atlas–initial_ were found to be of insufficient quality for training, we observed that their shortcomings can be mitigated by incorporating information about voxel intensities in the mask delineation process. This allowed us to formulate the following seven-step algorithm generating a corrected mask, X-mask_atlas–corrected_ from the X-mask_atlas–initial_:

1. Calculate the distribution of intensities of T1w image’s voxels within the initial mask, X-mask_atlas–initial_. Notably, apart from optic chiasm’s white matter voxels, this will include also false-positive voxels from adjacent tissue.
2. Calculate the 98th percentile of the obtained distribution. This threshold was identified empirically as the one resulting in the optimal separation of hyperintense voxels with blood vessel contributions from the surrounding.
3. Calculate the 66th percentile of the obtained distribution. This threshold was identified empirically as the one resulting in a robust and conservative separation of white matter voxels from partial-volume voxels and surrounding tissue.
4. Binarize a copy of the entire T1w image of the brain, setting all voxels to 0, except for those within the 66-98th percentile range.
5. From the binarized T1w image, extract a bounding box around the initial optic chiasm mask, extended by five voxels in left–right and posterior–anterior directions. This step is intended to exclude neighboring white matter structures which may interfere with step #6 and #7.
6. Extract the biggest cluster of nonzero voxels. This will represent the optic chiasm.
7. Dilate the cluster by one voxel in each direction. The conservatively chosen percentile thresholds, introduced in step #2 and #3, allowed extracting only non-surface voxels of optic chiasm, as they are affected by partial volume. As such, this step allows the possibility to include voxels at the surface.

The quantitative comparison (see *Computational methods*) of outcome for the X-mask_atlas–corrected_ with X-mask_manual_ demonstrated a significantly improved quality of the former (see *Results*) in comparison to X-mask_atlas–initial_. Given this validation, the correction procedure was subsequently performed for all of the HCP X-mask_atlas–initial_, and the resulting X-mask_atlas–corrected_ were used as targets for supervised training of the CNN.

### Convolutional Neural Network

This section describes in detail the architecture of the tested CNN, as well as the data preprocessing steps prior to training, training itself, and postprocessing of the output.

#### Network’s Architecture

The developed CNN used a 3D version (Çiçek et al., 2016) of the U-Net architecture (Ronneberger et al., 2015). Although the 3D version involves a higher computational load which may limit the upper resolution of processed images, the inclusion of additional dimension was shown to be of significant benefit to the segmentation (Chen et al., 2019; Mlynarski et al., 2020). Another reason for using the U-Net architecture was its reported robustness to jagged boundary-localized errors (Heller et al., 2018), which is a helpful feature in case of training on automatically generated masks.

Specifically, the network consists of analysis (encoding) and synthesis (decoding) paths. The analysis path contains four layers, each containing a standard U-net block [two 3 × 3 × 3 × convolutions followed by batch normalization and rectified linear unit (ReLu)] and subsequent 2 × 2 × 2 max pooling (stride of 2). For each subsequent step in the analysis path, the number of feature maps derived from input was doubled in each layer. The synthesis path consists of 2 × 2 × 2 upconvolution followed by a U-net block. Importantly, each decoding layer receives concatenated feature maps from a previous decoding layer and corresponding encoding layer, which allows for preservation of both low- and high-level features. Finally, in the last layer, the two output feature maps (background and target class, here optic chiasm) are being normalized with a voxelwise softmax function. The total number of parameters is 2,206,482.

#### Data Augmentation

Prior to being fed into the network, the training images and target X-mask_atlas–corrected_ were subjected to the following data augmentation procedure [performed using the TorchIO package (Pérez-García et al., 2021)], respectively:

• Normalization of maximal voxel intensity to 1. This adjusts for varied ranges of intensities between MR images originating from different sources, by rescaling intensities to 0-1 range.
• Random flip along any axis. This accounts for variations in the coordinate systems used for storing MRI images, such as in case of radiological and neurological conventions.
• Random crop to 160 × 160 × 160. This allows for generalization on the incomplete whole-brain data and eases the computational load.
• Random affine (rotation up to 15°, translation up to 20 voxels, bspline interpolation). This accounts for varied orientations and positions of the patient’s brain in the scanner space.

Performing these steps on the training data allowed us to enhance CNN’s generalization capabilities without resorting to explicit regularization, which has been demonstrated to limit the model’s capacity (Hernández-García and König, 2018). This was particularly important considering the misalignment of HCP training images (stored in MNI152 space) and CHIASM testing images (aligned to ACPC space).

#### Training and Loss Function

The HCP grayscale T1w images (*n* = 1049) and corresponding X-mask_atlas–corrected_ were divided into training (*n* = 932, 87.5%; HCP training—controls), validation (*n* = 107, 10%; HCP validation—controls), and testing (*n* = 10, 2.5%) subgroups augmented and subsequently fed in batches of 2 to U-Net CNN using the Dice similarity coefficient (see *Computational methods*) loss function and Adam optimizer (Kingma and Ba, 2017) for the purpose of weight updating. The training was performed five times, using different combinations of hyperparameters, specifically the maximal numbers of epochs (13, 15, 30, 40, 100) and learning rates (respectively, 0.0025, 0.0030, 0.0025, 0.0015, and 0.0005). The resulting weights of trained networks were saved and are provided (see *Availability of data and material*).

#### X-mask_CNN_

The trained CNN returned a grayscale image of the input’s size (160 × 160 × 160), where each voxel’s intensity depicted the probability of belonging to the optic chiasm (ranging from 0 to 1). This output was turned into a binary optic chiasm mask by thresholding the image to an empirically selected value (here we tested a range of 0.25, 0.50, 0.75, and 1.00 thresholds) and selecting the biggest cluster of nonzero elements present in the image. The quality of final X-mask_CNN_ generated by the tested range of training hyperparameters and threshold values were evaluated against X-mask_manual_ for both HCP test-controls and CHIASM controls datasets (see Supplementary Material), with the best-reported performance achieved for 30 epochs, and a learning rate equal to 0.0025 at a threshold of 1.

### Computational Methods

The employed computational methods incorporate the quantitative comparison of overlap of two masks by means of the Dice similarity coefficient (specified below), testing of mean equality using *t*-tests, and a range of classification metrics describing the discrepancy in results.

#### Dice Similarity Coefficient

In order to measure the quality of optic chiasm masks, we employed the Dice similarity coefficient [DSC; (Dice, 1945; Sørensen, 1948)] statistic, which describes the amount of overlap between two masks, in our case. The DSC ranges from 0 (lack of overlap) to 1 (perfect overlap of identical shapes). Specifically, we calculated the value of DSC between the ground-truth X-mask_manual_ and the candidate X-mask_‘candidate’_, where the latter has been previously limited only to axial slices where X-mask_manual_ was present (the excessive voxels were cropped). For brevity, the value of DSC calculated between X-mask_manual_ and candidate X-mask_‘candidate’_ is further being denoted to as DSC_manual_vs_‘candidate’_, and in case of group-level results, statistics is presented as mean ± standard error of mean (SEM).

#### Statistical Comparisons

The obtained DSC values, grouped with respect to compared candidate mask group (X-mask_initial_, X-mask_atlas–corrected_, and X-mask_CNN_) and participant group (HCP test-controls, CHIASM controls, and CHIASM albinism), were subjected to statistical testing. All samples were tested for normal distribution using the test by D’Agostino and Pearson (D’Agostino and Pearson, 1973), and for all but one (X-mask_atlas–initial_ : HCP test-controls) the null hypothesis of coming from normal distribution could not be rejected. Accordingly, in case of comparison of two normally distributed samples, we used two-tailed, two-sampled *t*-test at an alpha level of 5%; otherwise, we used the Wilcoxon rank-sum test, which tests the null hypothesis that two samples are drawn from the same distribution. Finally, we controlled for the familywise error (FWE) rate by applying Bonferroni’s correction to all calculated p-values.

#### Classification Metrics

In order to evaluate any potential discrepancy in the X-mask quality obtained for PWA and controls, we classified the obtained DSC_manual_vs_CNN_ using C-support vector classification (C-SVC) model with polynomial kernel (Platt, 1999; Chang and Lin, 2011). The measure of interclass discrepancy was subsequently quantified using well-established machine learning classification metrics, specifically

•
$Accuracy=\frac{TP+TN}{TP+TN+FP+FN}$

•
$Precision=\frac{TP}{TP+FP}$

•
$Recall\left(sensitivity\right)=\frac{TP}{TP+FN}$

•
$Specificity=\frac{TN}{TN+FP}$

where TP, TN, FP, and FN are, respectively, true positives, true negatives, false positives, and false negatives. Importantly, it should be noted that the classifier has been trained and evaluated on the same data, which is a clearly forbidden practice in case of evaluating a classifiers’ performance. Our goal was, however, to quantitatively express the intergroup differences in DSC_manual_vs_CNN_ and the overlap in data points, which is why we decided on such an approach. In line with that purpose, we used the support vector classification model which attempts to maximize the margin around the decision boundary.

## Results

This section provides a detailed qualitative and quantitative insight into the two key aspects of our investigation: (i) quality assessment of X-mask_atlas–initial_ and X-mask_atlas–corrected_ and (ii) evaluation of the CNN’s performance on the CHIASM dataset. An overview of the quantitative results is given in Table 1.

**TABLE 1:**
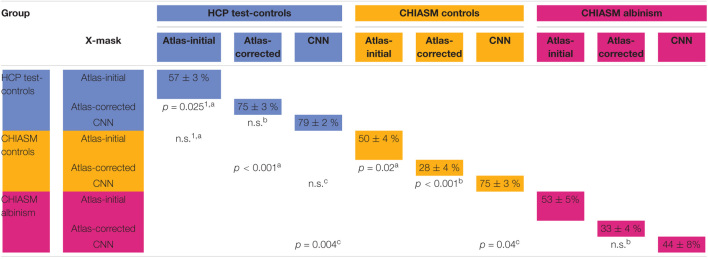
Mean and standard error of mean of DSC of X-mask and significance of cross-group differences.

*The diagonal displays the values of DSC of a specific X-mask (atlas-initial, atlas-corrected, CNN) compared to the corresponding X-mask_manual_ (0%: no overlap; 100%: identical masks, i.e., complete overlap) for each of the test groups (10 HCP test-controls, 8 CHIASM controls, and 9 CHIASM PWA). A total of 10 specific statistical tests were performed (corrected for familywise error using Bonferroni’s correction): four tests for cross-comparison of quality of X-mask_atlas–initial_ and X-mask_atlas–corrected_ for two control groups (marked by symbol ^a^), three tests for comparison of X-mask_atlas–corrected_ with X-mask_CNN_ for all groups (marked by symbol ^b^), and three tests for cross-comparisons of X-mask_CNN_ for all groups (marked by symbol ^c^). The p-values of the tests (either Wilcoxon rank-sum tests marked by symbol ^1^, or t-tests) comparing group DSC scores are displayed on the intersection of respective rows and columns (non-significant: n.s.; absence of test: blank cell). Blue color – HCP test-controls, yellow color – CHIASM controls, red color – CHIASM albinism.*

*^1^Wilcoxon rank-sum tests.*

*^a^Tests for cross-comparison of quality of X-mask_atlas–initial_ and X-mask_atlas–corrected_ for two control groups.*

*^b^Tests for comparison of X-mask_atlas–corrected_ with X-mask_CNN_ for all groups.*

*^c^Tests for cross-comparisons of X-mask_CNN_ for all groups.*

### Quality of Optic Chiasm Masks

**FIGURE 3:**
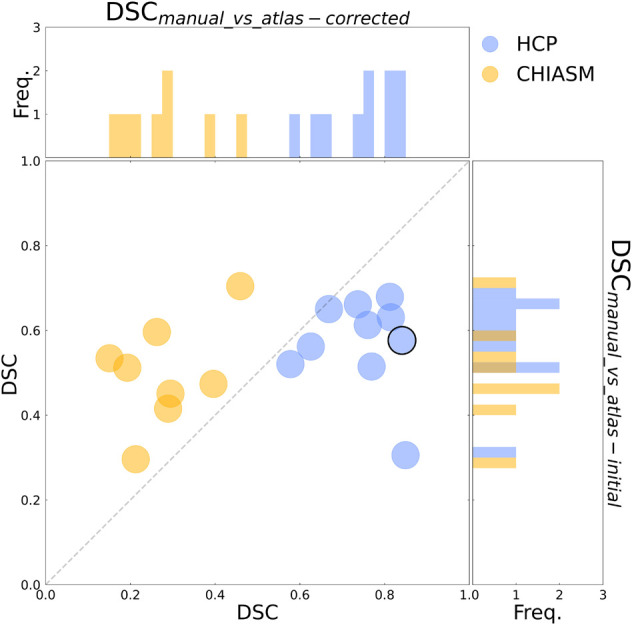
Evaluation of the mask correction procedure. Scatter plot of DSC_manual_vs_atlas–corrected_ (x-axis) and DSC_manual_vs_atlas–initial_ (y-axis) values for a subset of 10 HCP test-controls and 8 controls from the CHIASM dataset. The values are additionally presented in the form of marginal histograms of the two distributions—DSC_manual_vs_atlas–corrected_ (top horizontal) and DSC_manual_vs_atlas–initial_ (right vertical). The black contour marks the data point of the representative participant detailed in Figure 1.

As detailed in *Methods*, the quality of a candidate mask is determined based on its conformance with the ground-truth masks, i.e., DSC_manual_vs_‘candidate’_. The DSC_manual_vs_atlas–initial_ calculated for the 10 HCP test-controls was equal to 57 ± 3% (mean ± SEM). Upon correction of the X-mask_atlas–initial_ with the custom-designed algorithm, the quality of the corrected masks (X-mask_atlas–corrected_) improved significantly (mean DSC_manual_vs_atlas–initial_ and DSC_manual_vs_atlas–corrected_: 57 ± 3% and 75 ± 3%, respectively, FWE corrected p-value = 0.025; see also Figures 2, 3 for a quantitative and qualitative account, respectively). These results for the HCP test-controls provide support for the custom mask correction procedure, and as such X-mask_atlas–corrected_. For this reason of better quality, X-mask_atlas–corrected_ were later used for the CNN training.

Although the CNN training was based only on HCP data, the mask correction algorithm was tested also on the CHIASM dataset. The quality of X-mask_atlas–initial_ (DSC_manual_vs_atlas–initial_) of the CHIASM controls compares similarly to the HCP test-controls [HCP vs. CHIASM: 57 ± 3% vs. 50 ± 4 (mean ± SEM); p-value = 1.00]. In contrast, applying the mask correction procedure on the CHIASM data resulted in significantly lower DSC-measures for X-mask_atlas–corrected_ than X-mask_atlas–initial_ (DSC_manual_vs_atlas–initial_ vs. DSC_manual_vs_atlas–corrected_: 50 ± 4% vs. 28 ± 4%; *p*-value = 0.02; see Figure 3). Despite a comparable quality of initial masks, the quality of X-mask_atlas–corrected_ from the CHIASM dataset was reduced compared to the HCP X-mask_atlas–corrected_ [DSC_manual_vs_atlas_corrected_ (HCP vs. CHIASM): 75 ± 3% vs. 28 ± 4%; *p*-value < 0.001]. The findings reveal the limited generalization of the custom mask correction procedure. It should be noted that this is not of relevance for the hypothesis tested in this study: during training, the CNN is interacting with data and target masks corresponding to the HCP dataset only. Accordingly, while it is critical to ensure the high quality of training X-mask_atlas–corrected_, the CNN itself is agnostic to their derivation process and its limitations on other datasets. This will be proven further in the *Results* section (see *Transferability of CNNs*) where it will be shown that X-mask_atlas–corrected_ and X-mask_CNN_ of CHIASM controls are fundamentally different.

### Evaluation of Convolutional Neural Network’s Performance on the Testing Data

We calculated the DSC_manual_vs_CNN_ of control (*n* = 8) and PWA (*n* = 9) from the CHIASM dataset and HCP test-controls. This allowed us to gain insight into the (i) transferability of CNNs (i.e., how well the CNN performs on data from entirely new sources; for this purpose, we compared the quality of X-mask_atlas–corrected_ to X-mask_CNN_) and (ii) differences between optic chiasm masks computed by the CNN for controls and PWA.

**FIGURE 4:**
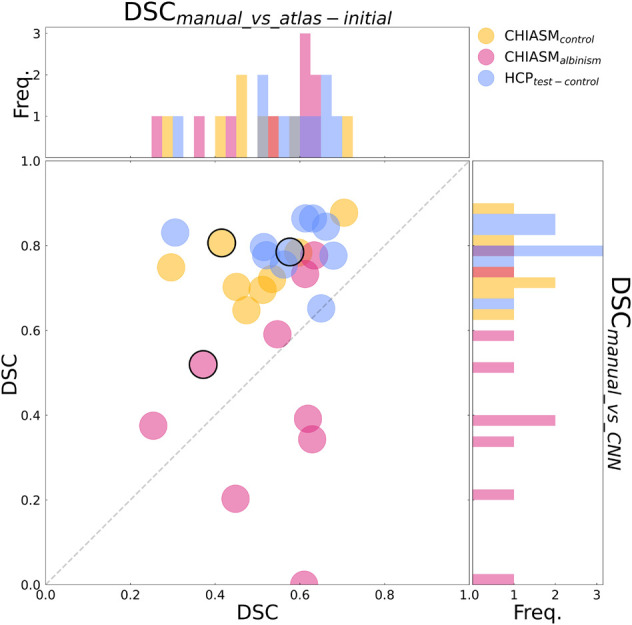
Comparison of quality of X-mask_atlas–initial_ and X-mask_CNN_. Scatter plot of DSC_manual_vs_atlas–initial_ (x-axis) and DSC_manual_vs_CNN_ (y-axis) values for CHIASM albinism (*n* = 9), CHIASM controls (*n* = 8), and HCP test-controls (*n* = 10). The values are additionally presented in the form of marginal histograms of the two distributions—DSC_manual_vs_atlas–initial_ (top horizontal) and DSC_manual_vs_CNN_ (right vertical). The black contours mark the data point of the representative participants, depicted in Figure 5. The outlier data point indicating DSC_manual_vs_CNN_ = 0 corresponds to a single case, where the chiasm could not be correctly identified. Specifically, X-mask_CNN_ was defined as a largest cluster of voxels with positive predictions (as output by CNN). In this unique case, however, the largest cluster was located outside the chiasm, in the cerebellum.

**FIGURE 5:**
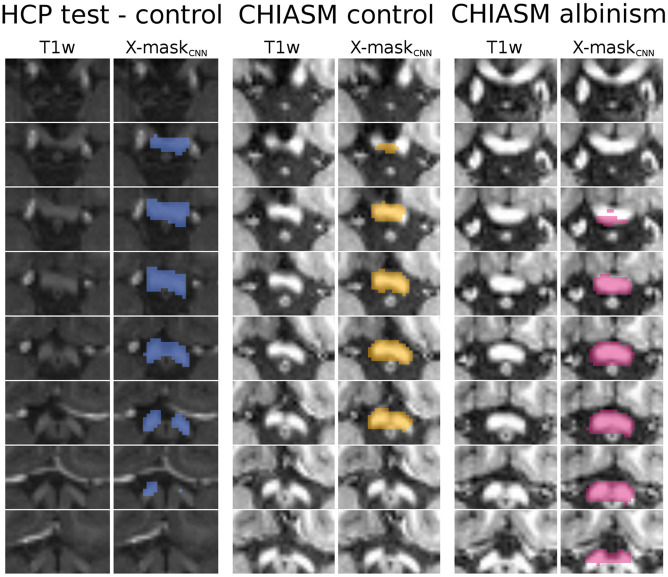
Overview of X-mask_CNN_ from a representative HCP test-control, CHIASM control, and CHIASM albinism participant. Axial slices display the optic chiasm region on the T1w image; blue-, yellow-, and magenta-colored masks show the X-mask_CNN_ defined for the HCP test-control, CHIASM control, and CHIASM albinism participants, respectively (marked by a black edge on Figure 4).

(i) Transferability of CNNs. The comparison of DSC_manual_vs_atlas–corrected_ with DSC_manual_vs_CNN_ performed for the 10 HCP test-controls failed to reveal a statistically significant difference [75 ± 3% and 79 ± 2%, respectively, *p*-value = 1.00]. This considerably deviated from results obtained for the CHIASM controls, where DSC_manual_vs_CNN_ (75 ± 3%) was significantly higher than DSC_manual_vs_atlas–corrected_ (28 ± 4%, *p*-value < 0.001). Interestingly, the values of DSC_manual_vs_CNN_ for both HCP test-controls and CHIASM controls were similar (79 ± 2% and 75 ± 3%, respectively; *p*-value = 1.00). The robust performance of the CNN on the CHIASM dataset reinforces the argument that CNN is agnostic to and does not copy the correction algorithm that generated training X-mask_atlas–corrected_ data (which was shown to fail on the data of CHIASM controls), but rather uses more general and robust processing that is well transferable to datasets different from the training one.
(ii) CNN-computed masks for controls vs. albinism. Comparing the DSC_manual_vs_CNN_ between CHIASM controls (75 ± 3%) and albinism participants (44 ± 8%) revealed a significantly lower quality of the latter (*p*-value = 0.04). These results also applied when substituting the CHIASM controls with the HCP test-controls (79 ± 2%, *p*-value = 0.004). An overview of the results is displayed in Figures 4, 5.

The observed differentiation between the controls and albinism was further investigated by measuring the performance of a C-SVC model (see *Methods*) applied to CHIASM albinism—CHIASM controls and CHIASM albinism—HCP test-controls data pairs (with PWA as positives and controls as negatives). The results of classification were subsequently evaluated with the metrics specified in *Methods* and detailed in Table 2.

**TABLE 2:** Accuracy of cross-group classification based on DSC_manual_vs_CNN_.

Metrics	CHIASM albinism vs. CHIASM controls	CHIASM albinism vs. HCP test-controls
Accuracy	0.89	0.84
Precision	0.80	0.82
Recall (sensitivity)	1.00	0.9
Specificity	0.78	0.78

The observed discrepancy in values of DSC_manual_vs_CNN_ for controls and albinism indicates that malformed chiasms are ill-represented by models “learned” from normal chiasms. This leads to the conclusion that both types of chiasms are described by diverse spatial features. This important observation provides a proof of concept for CNN-based direct classification of chiasms with regard to misrouting. At the same time, it should be noted that due to the limited sample of testing data, it is beyond the scope of the present study to provide an optimal DSC threshold for distinguishing controls from PWA. Given the clinical relevance of a threshold, we emphasize the need for future research to estimate this value through extensive evaluation of multiple datasets from several sources. While such a study is expected to provide the value for the optimal decision boundary (e.g., *via* a receiver operating characteristic curve analysis), the final proposed value should also factorize the consequences of type I and type II errors.

## Discussion

We aimed to investigate whether normal optic chiasms and those with misrouting as in albinism are represented by CNNs differently and, if yes, whether such differences could be used in the diagnostics of abnormal chiasms. In order to investigate this, we built and trained a CNN to segment optic chiasms based on control T1w images and algorithm-generated training masks. Our findings of a differential performance of the CNN in predicting normal and abnormal chiasms indeed highlight a potential utility of CNNs in identifying patients with chiasmal abnormalities. In the context of our findings, we intend to discuss two key aspects of our study: (i) use of automated masks in the training of CNN and (ii) application of control chiasms learned by CNN to abnormal cases.

### Use of Automated Masks in the Training of CNN

Automatically generated algorithm-based masks are bound to be a suboptimal solution for the purpose of image segmentation as compared to manual segmentations, specifically in medical images. Remarkably, we observed that in the case of a dataset with a specific structure, tailored adjustments can significantly increase the fidelity of automated masks to the ground truth. This strategy is useful in mitigating the disadvantages of a trade-off between mask quality and big sample size, as encountered when using automatically generated training data (McClure et al., 2019). This is further reinforced by the robust performance of the trained CNN here, demonstrating that the approximate masks despite individual flaws allow for a successful capture of the structure’s properties by the neural networks (Heller et al., 2018).

### Application of Control Chiasms Learned by Convolutional Neural Network to Abnormal Cases

Finally, the reported results demonstrated that the features of chiasms “learned” by training on control data does not apply to chiasms with enhanced misrouting, as present in albinism. This finding allows for a number of conclusions:

a. The chiasmal misrouting significantly alters the spatial organization of the optic chiasm [supporting the findings of Schmitz et al. (2003) and von dem Hagen et al. (2005)], which thus cannot be represented by data-driven models trained on control data only. Consequently, deep learning frameworks which exclude data of malformed chiasms from the training datasets will not be able to accurately represent them.
b. At the same time, the fundamental differences between CNN representations of normal and abnormal chiasms, as demonstrated by the quantified inaccuracy of masks, indicate the possibility of the identification of chiasmal misrouting from the T1w MRI images. Establishing such a method would provide direct and robust methods for the identification of misrouting in the clinical environment, which in turn is expected to reinforce the diagnostics of albinism. Further studies are needed to explore how our specific findings related to albinism translate to the detection of chiasma abnormalities in general.
c. The distinguished CNN representations of normal and abnormal chiasms are also a promising starting point for further studies addressing the association between chiasm malformations (with its further impact on white matter of visual system) and related reorganization at the level of the visual cortex (Hoffmann and Dumoulin, 2015). Additionally, the complexity of this phenomenon may greatly benefit from more complex methods, such as CNNs.
d. Finally, it should be noted that our current study underlines the general need for public datasets of rare, not infrequently overlooked, patient groups (Puzniak et al., in revision, see footnote 1). This is particularly important in the context of the current influx of deep learning-based tools in the healthcare system, such as CNNs trained to segment organs at risk (including chiasm as well) for therapy planning, which may not be available to rare patient groups not represented in training data. Considering this, the current study highlights the opportunities of improved diagnostics for the example of albinism and is intended to inspire the publication of further datasets to be utilized for the development of robust and transferable neural networks capable of accurate classification of chiasmal abnormalities from T1w MRI images.

### Limitations

The study limitations come from several distinct sources. Firstly, we note the limitations stemming from (i) lack of evidence on specific anatomical biomarkers of chiasmal malformations. Secondly, we acknowledge the limitations caused by data scarcity affecting the (ii) interpretability of the model, (iii) quantity and heterogeneity of the data, and (iv) quality of the data and labels. Finally, we note that the study might be partially affected by unavoidable (v) limitations in the design and training of the CNN.

#### Identification of Anatomical Biomarkers of Chiasmal Malformations

An important step in the validation of a diagnostic tool is the demonstration of its sensitivity to meaningful individualized biomarkers of the disease. Unfortunately, the evidence for such specific anatomical biomarkers of chiasmal malformations is missing. Although previous studies (Schmitz et al., 2003; von dem Hagen et al., 2005) provided a list of candidate features distinguishing normal from abnormal chiasms (width of chiasm, optic nerves and optic tracts, angle between optic tracts), none of these features were investigated in the context of individualized diagnostics. This lack of literature on the anatomy-based detection of chiasmal malformations was also the primary reason for the choice of our method here, which aimed at the investigation of the generalized applicability of CNNs for the purpose of detection of chiasmal malformations.

#### Interpretability of the Convolutional Neural Network

While the missing knowledge on specific relevant anatomical biomarkers of chiasmal misrouting can be retrieved by the identification of features driving the correct diagnostics, this process requires the CNN to be interpretable, i.e., to grant insight into identifying the input features driving the outcome decision. This property is, however, not generally available for all the CNNs but is rather dependent on their task. Specifically, in the case of described segmentation CNN (as chosen in this study to avoid dependence on the scarce data on chiasmal malformations), there are strong limits of the possibilities for visualization of the inference process. This limitation does not apply to the classification CNNs, where several interpretation techniques (Montavon et al., 2018) such as Grad-CAM (Selvaraju et al., 2017) can be implemented. These, however, require extensive datasets. Additionally, as this type of network uses both normal and abnormal data for training, it allows for extensive validation of features that drive the CNN’s decision that will lead to understand their placing in the normal–abnormal spectrum. In summary, we note that while the employed approach provides the evidence that certain anatomical biomarkers of chiasmal malformations exist, the next step should involve their identification with a classifying CNN trained on larger datasets comprising both normal and abnormal data.

#### Quantity and Heterogeneity of the Data

Although our study’s design enabled us to take advantage of the massive HCP dataset and train the CNN on much bigger samples than typically used, the rarity of albinism (and other cases of congenital malformations of the chiasm) severely limited the size of the training sample. For the same reasons, we were limited to testing data from only one site, which prevented us from investigation of impact of scanner and data acquisition protocol on the method’s outcome. Moreover, this limited our estimates of the accuracy and robustness of the presented method.

#### Quality of Data and Labels

Although the HCP dataset is well-known for setting standards in MRI data quality assessment, it does not contain any clinical information pertaining to the participants’ visual system evaluations. Furthermore, the training dataset might include participants with retinal and/or optic nerve disorders in proportions corresponding to their representation in the real world. This prevalence is, however, not expected to influence the outcome of CNN training, but even if so, this would cause underestimation of our method’s performance, rather than overestimation.

Similarly, although the CHIASM datasets provides findings of ophthalmologic examination of included participants, it does not provide information about the types of albinism represented in the dataset (e.g., oculo-cutaneous, ocular albinism).

Finally, the quality of automatically generated training labels is inferior to the ones created manually. Although we provide evidence that this does not impact the general outcome of the study, we acknowledge the use of manually defined labels to be the optimal approach.

#### Convolutional Neural Network Design and Training

Due to the models’ complexity and high dependence on the underlying data, the Deep Learning modeling approach is mainly driven by empirical, rather than theoretical, evidence. Consequently, despite the choice of employing an established 3D U-Net architecture, which was reported to perform well in similar task, we cannot rule out that other architectures would not provide better results. Similarly during CNN training, although we tried several combinations of hyperparameters and reported the ones yielding the best results, it is nearly impossible that we found the globally optimal configuration of the CNN network’s parameters.

## Data Availability Statement

The datasets presented in this study can be found in online repositories. Specifically, this includes:

- T1w images from the HCP dataset stored on the brainlife.io platform: https://brainlife.io/project/5941a225f876b000210c11e5
- T1w images from the CHIASM dataset stored on the brainlife.io platform: https://brainlife.io/pub/5dea42a96c0bd9c0508554a2
- X-mask_manual_, X-mask_atlas–initial_, X-mask_atlas–corrected_, and X-mask_CNN_ stored on the brainlife.io platform: https://brainlife.io/project/6043cb8966d5ce5fc26f5f73
- The weights of the trained network stored on osf.io platform: https://osf.io/4cvgq/
  The code used in this study for generation of training data, CNN training, analyses, and figures can be found on github.com platform: https://github.com/rjpuzniak/Use-of-deep-learning-based-optic-chiasm-segmentation-for-investigating-visual-system-pathophysiology.

## Ethics Statement

Ethical review and approval was not required for the study on human participants in accordance with the local legislation and institutional requirements. Written informed consent for participation was not required for this study in accordance with the national legislation and the institutional requirements.

## Author Contributions

RP and MH designed the study and interpreted the data. RP performed the analysis. RP, GP, and MH wrote the manuscript. All authors contributed to the article and approved the submitted version.

## Conflict of Interest

The authors declare that the research was conducted in the absence of any commercial or financial relationships that could be construed as a potential conflict of interest.

## Publisher’s Note

All claims expressed in this article are solely those of the authors and do not necessarily represent those of their affiliated organizations, or those of the publisher, the editors and the reviewers. Any product that may be evaluated in this article, or claim that may be made by its manufacturer, is not guaranteed or endorsed by the publisher.

## Footnotes

1 Puzniak, R. J., et al. (in revision). CHIASM, The Human Brain Albinism and Achiasma MRI Dataset.

2
https://brainlife.io/project/5941a225f876b000210c11e5

3
https://brainlife.io/pub/5dea42a96c0bd9c0508554a2

## References

[B1] Ahmadi K. Herbik A. Wagner M. Kanowski M. Thieme H. Hoffmann M. B. (2019). Population receptive field and connectivity properties of the early visual cortex in human albinism. *Neuroimage* 202:116105. 10.1016/j.neuroimage.2019.116105 31422172

[B2] Ather S. Proudlock F. A. Welton T. Morgan P. S. Sheth V. Gottlob I. (2018). Aberrant visual pathway development in albinism: from retina to cortex. *Hum. Brain Mapp.* 40 777–788. 10.1002/hbm.24411 30511784 PMC6865554

[B3] Avesani P. McPherson B. Hayashi S. Caiafa C. F. Henschel R. Garyfallidis E. (2019). The open diffusion data derivatives, brain data upcycling via integrated publishing of derivatives and reproducible open cloud services. *Sci. Data* 6:69. 10.1038/s41597-019-0073-y 31123325 PMC6533280

[B4] Brodsky M. C. Glasier C. M. Creel D. J. (1993). Magnetic resonance imaging of the visual pathways in human albinos. *J. Pediatr. Ophthalmol. Strabismus* 30 382–385. 8120744 10.3928/0191-3913-19931101-09

[B5] Chang C.-C. Lin C.-J. (2011). LIBSVM: a library for support vector machines. *ACM Trans. Intell. Syst. Technol.* 2 1–27. 10.1145/1961189.1961199

[B6] Chen H. Lu W. Chen M. Zhou L. Timmerman R. Tu D. (2019). A recursive ensemble organ segmentation (REOS) framework: application in brain radiotherapy. *Phys. Med. Biol.* 64:025015. 10.1088/1361-6560/aaf83c 30540975

[B7] Çiçek Ö Abdulkadir A. Lienkamp S. S. Brox T. Ronneberger O. (2016). “D U-Net: learning dense volumetric segmentation from sparse annotation,” in *Medical Image Computing and Computer-Assisted Intervention–MICCAI 2016. (Lecture Notes in Computer Science)*, eds Ourselin S. Joskowicz L. Sabuncu M. Unal G. Wells W. (Cham: Springer), 424–432. 10.1007/978-3-319-46723-8_49

[B8] D’Agostino R. Pearson E. S. (1973). Tests for departure from normality. Empirical results for the distributions of b2 and ✓ b1. *Biometrika* 60 613–622. 10.2307/2335012

[B9] Dice L. R. (1945). Measures of the amount of ecologic association between species. *Ecology* 26 297–302. 10.2307/1932409

[B10] Duanmu H. Kim J. Kanakaraj P. Wang A. Joshua J. Kong J. (2020). “Automatic brain organ segmentation with 3D fully convolutional neural network for radiation therapy treatment planning,” in *Proceedings of the 2020 IEEE 17th International Symposium on Biomedical Imaging (ISBI)*, Iowa City, IA, 758–762. 10.1109/ISBI45749.2020.9098485 PMC7427623 32802270

[B11] Fedorov A. Damaraju E. Calhoun V. Plis S. (2017a). Almost instant brain atlas segmentation for large-scale studies. *arXiv* [Preprint]. Available online at: http://arxiv.org/abs/1711.00457 (accessed March 16, 2021). arXiv:1711.00457 [cs],

[B12] Fedorov A. Johnson J. Damaraju E. Ozerin A. Calhoun V. Plis S. (2017b). “End-to-end learning of brain tissue segmentation from imperfect labeling,” in *Proceedings of the 2017 International Joint Conference on Neural Networks (IJCNN)*, Anchorage, AK, 3785–3792. 10.1109/IJCNN.2017.7966333

[B13] Fischl B. (2012). FreeSurfer. *Neuroimage* 62 774–781. 10.1016/j.neuroimage.2012.01.021 22248573 PMC3685476

[B14] Glasser M. F. Sotiropoulos S. N. Wilson J. A. Coalson T. S. Fischl B. Andersson J. L. (2013). The minimal preprocessing pipelines for the human connectome project. *Neuroimage* 80 105–124. 10.1016/j.neuroimage.2013.04.127 23668970 PMC3720813

[B15] Heller N. Dean J. Papanikolopoulos N. (2018). “Imperfect segmentation labels: how much do they matter?,” in *Intravascular Imaging and Computer Assisted Stenting and Large-Scale Annotation of Biomedical Data and Expert Label Synthesis. (Lecture Notes in Computer Science)*, eds Stoyanov D. Taylor Z. Balocco S. Sznitman R. Martel A. Maier-Hein L. (Cham: Springer International Publishing), 112–120. 10.1007/978-3-030-01364-6_13

[B16] Hernández-García A. König P. (2018). Data augmentation instead of explicit regularization. *arXiv* [Preprint]. arXiv:1806.03852,

[B17] Hoffmann M. B. Dumoulin S. O. (2015). Congenital visual pathway abnormalities: a window onto cortical stability and plasticity. *Trends Neurosci.* 38 55–65. 10.1016/j.tins.2014.09.005 25448619

[B18] Hoffmann M. B. Lorenz B. Morland A. B. Schmidtborn L. C. (2005). Misrouting of the optic nerves in albinism: estimation of the extent with visual evoked potentials. *Invest. Ophthalmol. Vis. Sci.* 46 3892–3898. 10.1167/iovs.05-0491 16186379

[B19] Hoffmann M. B. Schmidtborn L. C. Morland A. B. (2007). Abnormale repräsentationen im visuellen kortex von albinismus-patienten. *Der Ophthalmol.* 104 666–673. 10.1007/s00347-007-1589-7 17661055

[B20] Hoffmann M. B. Tolhurst D. J. Moore A. T. Morland A. B. (2003). Organization of the visual cortex in human albinism. *J. Neurosci.* 23 8921–8930. 10.1523/JNEUROSCI.23-26-08921.2003 14523094 PMC6740392

[B21] Ibragimov B. Xing L. (2017). Segmentation of organs-at-risks in head and neck CT images using convolutional neural networks. *Med. Phys.* 44 547–557. 10.1002/mp.12045 28205307 PMC5383420

[B22] Kingma D. P. Ba J. (2017). Adam: a method for stochastic optimization. *arXiv* [Preprint]. Available online at: http://arxiv.org/abs/1412.6980 (accessed March 4 2021). arXiv:1412.6980 [cs]

[B23] Krizhevsky A. Sutskever I. Hinton G. E. (2012). *ImageNet Classification with Deep Convolutional Neural Networks, Advances in Neural Information Processing Systems, 25.* Available online at: https://proceedings.neurips.cc/paper/2012/file/c399862d3b9d6b76c8436e924a68c45b-Paper.pdf (accessed March 2 2021).

[B24] Kruijt C. C. de Wit G. C. Bergen A. A. Florijn R. J. Schalij-Delfos N. E. van Genderen M. M. (2018). The phenotypic spectrum of albinism. *Ophthalmology* 125 1953–1960. 10.1016/j.ophtha.2018.08.003 30098354

[B25] Kupfer C. Chumbley L. Downer J. C. (1967). Quantitative histology of optic nerve, optic tract and lateral geniculate nucleus of man. *J. Anat.* 101(Pt 3) 393–401. 6051727 PMC1270921

[B26] LeCun Y. Boser B. Denker J. S. Henderson D. Howard R. E. Hubbard W. (1989). Backpropagation applied to handwritten zip code recognition. *Neural Comput.* 1 541–551. 10.1162/neco.1989.1.4.541

[B27] Lundervold A. S. Lundervold A. (2019). An overview of deep learning in medical imaging focusing on MRI. *Z. Med. Phys.* 29 102–127. 10.1016/j.zemedi.2018.11.002 30553609

[B28] Marçon C. R. Maia M. (2019). Albinism: epidemiology, genetics, cutaneous characterization, psychosocial factors. *An. Bras. Dermatol.* 94 503–520. 10.1016/j.abd.2019.09.023 31777350 PMC6857599

[B29] McClure P. Rho N. Lee J. A. Kaczmarzyk J. R. Zheng C. Y. Ghosh S. S. (2019). Knowing what you know in brain segmentation using bayesian deep neural networks. *Front. Neuroinform.* 13:67. 10.3389/fninf.2019.00067 31749693 PMC6843052

[B30] Milchenko M. Marcus D. (2013). Obscuring surface anatomy in volumetric imaging data. *Neuroinformatics* 11 65–75. 10.1007/s12021-012-9160-3 22968671 PMC3538950

[B31] Mlynarski P. Delingette H. Alghamdi H. Bondiau P. Y. Ayache N. (2020). Anatomically consistent CNN-based segmentation of organs-at-risk in cranial radiotherapy. *J. Med. Imaging* 7:014502. 10.1117/1.JMI.7.1.014502 PMC7016364 32064300

[B32] Montavon G. Samek W. Müller K.-R. (2018). Methods for interpreting and understanding deep neural networks. *Digit. Signal Process.* 73 1–15. 10.1016/j.dsp.2017.10.011

[B33] Pérez-García F. Sparks R. Ourselin S. (2021). TorchIO: a Python library for efficient loading, preprocessing, augmentation and patch-based sampling of medical images in deep learning. *arXiv* [Preprint]. Available online at: http://arxiv.org/abs/2003.04696 (accessed 4 March 2021) arXiv:2003.04696 [cs, eess, stat] 10.1016/j.cmpb.2021.106236 PMC8542803 34311413

[B34] Platt J. C. (1999). “Probabilistic outputs for support vector machines and comparisons to regularized likelihood methods,” in *Advances in Large Margin Classifiers*, eds Smola A. J. Bartlett P. Schölkopf B. Schuurmans D. (Cambridge, MA: MIT Press), 61–74.

[B35] Puzniak R. J. Ahmadi K. Kaufmann J. Gouws A. Morland A. B. Pestilli F. (2019). Quantifying nerve decussation abnormalities in the optic chiasm. *Neuroimage Clin.* 24:102055. 10.1016/j.nicl.2019.102055 31722288 PMC6849426

[B36] Puzniak R. J. Prabhakaran G. T. Buentjen L. Schmitt F. C. Hoffmann M. B. (2021). Tracking the visual system—from the optic chiasm to primary visual cortex. *Z. Epileptol.* 34 57–66. 10.1007/s10309-020-00384-y

[B37] Rebsam A. Bhansali P. Mason C. A. (2012). Eye-specific projections of retinogeniculate axons are altered in albino mice. *J. Neurosci.* 32 4821–4826. 10.1523/JNEUROSCI.5050-11.2012 22492037 PMC3329942

[B38] Ronneberger O. Fischer P. Brox T. (2015). “U-Net: convolutional networks for biomedical image segmentation,” in *Medical Image Computing and Computer-Assisted Intervention–MICCAI 2015 (Lecture Notes in Computer Science)*, eds Navab N. Hornegger J. Wells W. Frangi A. (Cham: Springer International Publishing), 234–241. 10.1007/978-3-319-24574-4_28

[B39] Schmitz B. Schaefer T. Krick C. M. Reith W. Backens M. Käsmann-Kellner B. (2003). Configuration of the optic chiasm in humans with albinism as revealed by magnetic resonance imaging. *Invest. Ophthalmol. Vis. Sci.* 44 16–21. 10.1167/iovs.02-0156 12506050

[B40] Selvaraju R. R. Cogswell M. Das A. Vedantam R. Parikh D. Batra D. (2017). “Grad-CAM: visual explanations from deep networks via gradient-based localization,” in *Proceedigs of the 2017 IEEE International Conference on Computer Vision (ICCV)*, Venice, 618–626. 10.1109/ICCV.2017.74

[B41] Sørensen T. J. (1948). *A Method of Establishing Groups of Equal Amplitude in Plant Sociology Based on Similarity of Species Content and its Application to Analyses of the Vegetation on Danish Commons.* København: I kommission hos E. Munksgaard.

[B42] Tong N. Gou S. Yang S. Ruan D. Sheng K. (2018). Fully automatic multi-organ segmentation for head and neck cancer radiotherapy using shape representation model constrained fully convolutional neural networks. *Med. Phys.* 45 4558–4567. 10.1002/mp.13147 30136285 PMC6181786

[B43] van der Kouwe A. J. W. Benner T. Salat D. H. Fischl B. (2008). Brain morphometry with multiecho MPRAGE. *Neuroimage* 40 559–569. 10.1016/j.neuroimage.2007.12.025 18242102 PMC2408694

[B44] Van Essen D. C. Smith S. M. Barch D. M. Behrens T. E. J. Yacoub E. Uğurbil K. (2013). The wu-minn human connectome project: an overview. *Neuroimage* 80 62–79. 10.1016/j.neuroimage.2013.05.041 23684880 PMC3724347

[B45] von dem Hagen E. A. H. Hoffmann M. B. Morland A. B. (2008). Identifying human albinism: a comparison of VEP and fMRI. *Invest. Ophthalmol. Vis. Sci.* 49 238–249. 10.1167/iovs.07-0458 18172098

[B46] von dem Hagen E. A. H. Houston G. C. Hoffmann M. B. Jeffery G. Morland A. B. (2005). Retinal abnormalities in human albinism translate into a reduction of grey matter in the occipital cortex. *Eur. J. Neurosci.* 22 2475–2480. 10.1111/j.1460-9568.2005.04433.x 16307590

[B47] Yao A. D. Cheng D. L. Pan I. Kitamura F. (2020). Deep learning in neuroradiology: a systematic review of current algorithms and approaches for the new wave of imaging technology. *Radiol. Artif. Intell.* 2:e190026. 10.1148/ryai.2020190026 33937816 PMC8017426

[B48] Zhu W. Huang Y. Zeng L. Chen X. Liu Y. Qian Z. (2019). AnatomyNet: deep learning for fast and fully automated whole-volume segmentation of head and neck anatomy. *Med. Phys.* 46 576–589. 10.1002/mp.13300 30480818

