# Supplementary material for: Deep Learning-Based Detection of Malformed Optic Chiasms From MRI Images
Source: Front Neurosci. 2021 Oct 25;15:755785. doi: 10.3389/fnins.2021.755785 (PMC8573410; doi:10.3389/fnins.2021.755785)
Supplement: Supplementary file 2 [file Table_2.docx]

**Supplementary Table 2.** Mean DSC_manual_vs­_CNN_ (mean ± SEM) for CHIASM data (controls). EP and LR stands for epochs and learning rate, respectively. Thresholds depict the cut-off values used to transform the output image of CNN into binarized X-mask_CNN._

| **Hyperparameters** | **Threshold 0.25** | **Threshold 0.50** | **Threshold 0.75** | **Threshold 1.00** |
| --- | --- | --- | --- | --- |
| 13 EP 0.0025 LR | 0.58 ± 0.04 | 0.57 ± 0.4 | 0.55 ± 0.4 | 0.25 ± 0.06 |
| 15 EP 0.0030 LR | 0.10 ± 0.03 | 0.09 ± 0.03 | 0.08 ± 0.02 | 0.03 ± 0.01 |
| 30 EP 0.0025 LR | 0.78 ± 0.03 | 0.78 ± 0.03 | 0.77 ± 0.03 | 0.75 ± 0.03 |
| 40 EP 0.0015 LR | 0.60 ± 0.03 | 0.60 ± 0.03 | 0.60 ± 0.03 | 0.43 ± 0.04 |
| 100 EP 0.0005 LR | 0.40 ± 0.05 | 0.39 ± 0.05 | 0.38 ± 0.05 | 0.26 ± 0.05 |
